# A Case of Hyperacute Severe Thrombocytopenia Occurring Less than 24 Hours after Intravenous Tirofiban Infusion

**DOI:** 10.1155/2018/4357981

**Published:** 2018-05-24

**Authors:** Vineet Meghrajani, Nitin Sabharwal, Vinod Namana, Moustafa Elsheshtawy, Bernard Topi

**Affiliations:** ^1^Department of Internal Medicine, Maimonides Medical Center, 4802 10th Avenue, Brooklyn, NY 11219, USA; ^2^Department of Cardiology, Maimonides Medical Center, 4802 10th Avenue, Brooklyn, NY 11219, USA

## Abstract

Thrombocytopenia is defined as a condition where the platelet count is below the lower limit of normal (<150 G/L), and it is categorized as mild (100–149 G/L), moderate (50–99 G/L), and severe (<50 G/L). We present here a 79-year-old man who developed severe thrombocytopenia with a platelet count of 6 G/L, less than 24 hours after intravenous tirofiban infusion that was given to the patient during a percutaneous transluminal coronary angioplasty procedure with placement of 3 drug-eluting stents. The patient's baseline platelet count was 233 G/L before the procedure. Based on the timeline of events during hospitalization and laboratory evidence, it was highly likely that the patient's thrombocytopenia was the result of tirofiban-induced immune thrombocytopenia, a type of drug-induced immune thrombocytopenia (DITP) which occurs due to drug-dependent antibody-mediated platelet destruction. Anticoagulant-mediated artefactual pseudothrombocytopenia was ruled out as no platelet clumping was seen on the peripheral blood smears. The treatment of DITP includes discontinuation of the causative drug; monitoring of platelet count recovery; or treatment of severe thrombocytopenia with glucocorticoids, IVIG, or platelet transfusions depending on the clinical presentation. The most likely causative agent of this patient's thrombocytopenia—tirofiban—was discontinued, and the patient did not develop any signs of bleeding during the remainder of his hospital stay. His platelet count gradually improved to 24 G/L, and he was discharged on the sixth hospital day.

## 1. Case

**Figure 1:**
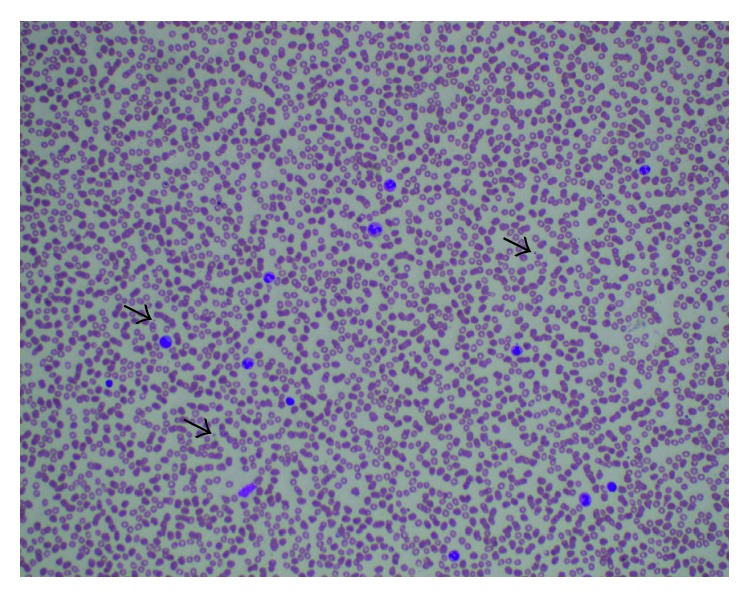
A Wright–Giemsa stained peripheral blood film of the patient under 200x microscopic magnification showing absence of platelet clumping. Scattered platelets are seen next to blue arrows with no clumping.

A 79-year-old independently functioning man presented to the emergency room with an episode of substernal chest pain. His medical history included stable coronary artery disease, chronic systolic congestive heart failure with a left ventricular ejection fraction of 25%, paroxysmal atrial fibrillation, type 2 diabetes mellitus, hyperlipidemia, and hypertension. The patient also had a cardiac resynchronization therapy defibrillator (CRT-D) device in place for 5 years. He described the chest pain as a sensation of substernal crushing that started when he was resting comfortably at home. The pain was moderate in intensity, nonradiating, and lasted for approximately 10 minutes before resolving spontaneously. He denied any dyspnea, palpitations, dizziness, or loss of consciousness during this episode. He had never smoked before. His home medications included aspirin 81 mg daily, losartan 50 mg daily, carvedilol 6.25 mg twice daily, apixaban 5 mg twice daily, and furosemide 40 mg daily. On arrival in the emergency room, the patient was asymptomatic and hemodynamically stable. Electrocardiogram showed a paced cardiac rhythm with no acute ST segment or T wave changes. Laboratory workup sent on arrival to the ER included a complete blood count and coagulation profile with the following results: white blood cell count of 8.0 G/L, hemoglobin level of 14.2 g/dl, and platelet count of 232 G/L. The patient had an initial cardiac troponin level of 0.06 and a B-type natriuretic peptide level of 190 pg/ml. Follow-up cardiac troponin levels were 0.06 ng/ml and 0.05 ng/ml, respectively. Based on this presentation, it was decided to admit the patient to the telemetry floor for a possible NSTEMI with a TIMI score of 5, and treatment was initiated with intravenous unfractionated heparin drip at 12 units/kg/hour. Monitoring of the aPTT was done every 6 hours for a total of 12 hours after initiation of heparin drip and once daily thereafter, to target the dosing of heparin. Therapeutic anticoagulation levels of aPTTs (between 52.0 and 79.9 seconds) were achieved throughout the duration of treatment with heparin drip. The patient's apixaban was held. A persantine thallium cardiac stress test was ordered, which showed moderate inferoapical left ventricular wall ischemia and inferior wall defect. The patient was subsequently planned for an elective coronary angiogram, which showed 80%–90% stenoses of the middle, distal, and second diagonal segments of the left anterior descending coronary artery with no acute coronary artery occlusions. The patient underwent placement of 3 drug-eluting stents in the stenotic coronary segments during the procedure, received aspirin 325 mg, clopidogrel 600 mg orally, and a bolus dose of tirofiban (5 mg/100 ml) 42.5 ml intravenously and later the same day, was started on a tirofiban drip at a maintenance infusion rate of 0.15 mcg/kg/min for 3 hours. Used with PCI, glycoprotein (GP) IIb/IIIa inhibitors like tirofiban and abciximab have been shown to reduce the rates of death, myocardial infarction, and urgent target-vessel revascularization in patients with STEMI and NSTEMI. It was estimated that the patient had received a total of 89.5 ml (4.5 mg) of tirofiban intravenously on the day of the coronary angiogram including the bolus dose and the maintenance dose. The patient's platelet count on the automated complete blood count test (CBC) drawn at 7.52 am on the day of the coronary angiogram was 233 G/L, which dropped to 6 G/L on the automated CBC drawn at 6.53 am the day after the procedure. A repeat CBC was ordered at 11.50 am after the result of the first CBC drawn earlier during the day was reported, and it showed a platelet count of 10 G/L (Table 1). The CBC tests were run on blood collected from the patient in specimen tubes containing EDTA as an anticoagulant. The automated CBC tests were processed by using Beckman Coulter model LH 780 hematology blood analyzer, with the machine set to flag a result if the platelet count on a specimen was less than 50 G/L. The automated CBC tests did not have any flags reported on the analyzer for platelet clumping in all the CBC tests performed on the patient's blood. A platelet count was subsequently done by visual inspection of a hematological smear of the blood specimens under microscope, which confirmed the result of the automated CBC and did not show any platelet clumping (Figure 1).

**Figure 2:**
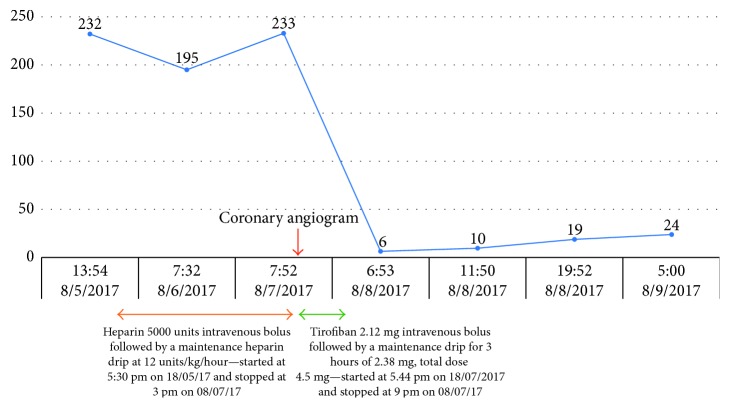
Platelet count in G/L over time with relation to drug exposure.

The patient did not have any signs of bleeding, including ecchymosis, bruises, hematuria, or blood in stool. He was subsequently admitted to the cardiac intensive care unit for further monitoring and all anticoagulation was held. Dual antiplatelet therapy with aspirin and clopidogrel was however continued, considering that he had recently undergone percutaneous coronary intervention with placement of drug-eluting stents and was therefore at a high risk of stent thrombosis. Another CBC drawn at 7.52 pm the same day showed improvement in the platelet count to 19 G/L. The patient did not develop any signs of bleeding during the remainder of his hospital stay, and his platelet count gradually improved to 24 G/L (Figure 2) before he was discharged on the sixth hospital day on aspirin and clopidogrel. The patient was provided outpatient follow-up with a plan to eventually resume anticoagulation with apixaban for paroxysmal atrial fibrillation once his platelet count had recovered to stable levels.

## 2. Discussion

Thrombocytopenia is defined as a condition where the platelet count is below the lower limit of normal (<150 G/L), and it is categorized as mild (100–149 G/L), moderate (50–99 G/L), and severe (<50 G/L) [1]. The primary differential diagnoses in consideration for this patient's unexplained thrombocytopenia included pseudothrombocytopenia, heparin-induced thrombocytopenia, and GP IIb/IIIa inhibitor- (tirofiban-) induced immune thrombocytopenia. Pseudothrombocytopenia is a term used to describe a falsely low-platelet count occurring due to a laboratory artifact, in which the anticoagulant EDTA used in specimen tubes may induce platelet clumping resulting in an artificially low-platelet count [2]. Pseudothrombocytopenia can be ruled out by visual inspection of the peripheral blood smear under microscope to check for platelet clumping and repeating the platelet count, both of which were done for our patient, with no clumping seen on the peripheral smear and a platelet count that remained low on a subsequently drawn CBC. Heparin-induced thrombocytopenia (HIT) is caused by autoantibodies to platelet factor 4 (PF4) complexed with heparin, which in turn activate platelets and cause widespread arterial and venous thrombosis and thrombocytopenia [3–6]. The “4Ts score” is a pretest scoring system for HIT that was developed to improve and standardize clinical diagnosis of heparin-induced thrombocytopenia [7–9] (Table 2). Although the patient had been receiving intravenous unfractioned heparin less than 24 hours before the drop in the platelet count, his 4Ts score was calculated to be 1, indicating that the probability of the patient having heparin-induced thrombocytopenia (HIT) was low (less than 5%). He had not received heparin in the 100 days prior to this hospitalization.

Based on the clinical presentation, the timeline of events during hospitalization and laboratory evidence, it was highly likely that the patient's thrombocytopenia was the result of tirofiban-induced immune thrombocytopenia, as evidenced by its rapid onset (<24 hours), and improvement in platelet count within a day of discontinuation of tirofiban. Glycoprotein IIb/IIIa receptor antagonists, including abciximab, eptifibatide, and tirofiban, are a class of antiplatelet medications that reduce platelet aggregation and thrombus formation through the blockade of key binding sites needed to stabilize the forming platelet aggregate [10]. Tirofiban is a small molecule reversible, competitive inhibitor of GP IIb/IIIa receptors that recognizes the arginine-glycine-aspartic acid- (RGD-) binding sequence in the *β*3 subunit of the GP IIb/IIIa receptor and binds within the ligand-binding pocket of the receptor to compete with fibrinogen, vWF, and other ligands resulting in inhibition of thrombi formation [11, 12]. GP IIb/IIIa antagonists are widely used in the management of patients with acute coronary syndromes and patients undergoing coronary artery stenting due to their effects of preventing platelet aggregation and thrombus formation [13]. Used with PCI, glycoprotein (GP) IIb/IIIa inhibitors have been shown to reduce the rates of death, myocardial infarction, and urgent target-vessel revascularization in patients with STEMI and NSTEMI [14]. A documented side effect of GP IIb/IIIa inhibitors is thrombocytopenia, the incidence of which is reported to be higher in those receiving abciximab compared to eptifibatide or tirofiban [15–26]. Thrombocytopenia occurring secondary to GP IIb/IIIa inhibitors is included in a category of thrombocytopenia called drug-induced immune thrombocytopenia (DITP) which occurs due to drug-dependent antibody-mediated platelet destruction [27, 28]. Drugs most commonly known to cause DITP include quinine, quinidine, trimethoprim-sulfamethoxazole, vancomycin, penicillin, rifampin, carbamazepine, ceftriaxone, ibuprofen, mirtazapine, oxaliplatin, suramin, GP IIb/IIIa inhibitors, and heparin (a comprehensive list can be obtained at http://www.ouhsc.edu/platelets/) [29, 30]. There are various mechanisms by which these drugs cause DITP, with the mechanism depending on the type of drug causing it. Drugs like quinine and various antibiotics act by binding to the complementarity-determining region (CDR) of the antibody and modify it so that it acquires the ability to bind to a platelet glycoprotein, usually GP Ib/IX or GP IIb/IIIa. These antibodies may (before affinity maturation) be derived from naturally occurring immunoglobulins that, in the absence of drug, bind very weakly to these targets and are clinically insignificant [31–33]. Abciximab-dependent antibodies appear to recognize mouse-derived protein elements present in the mostly humanized Fab drug or conformational epitopes (neoepitopes) induced in the abciximab molecule when it binds to GP IIb/IIIa [18]. Ligand-mimetic GP IIb/IIIa inhibitors like tirofiban and eptifibatide recognize neoepitopes induced in the GP IIb/IIIa head structure when these drugs react with the RGD recognition site and greatly increase the affinity of these antibodies for binding to platelet surface antigens by inducing structural changes in the GP IIb/IIIa receptor, thereby accelerating their destruction. Such antibodies may be naturally occurring, which may explain the observation of extremely rapid platelet count drop with GP IIb/IIIa inhibitors [15, 32, 34–37]. DITP is reversible upon drug discontinuation, with the platelet count expected to increase within one to two days of drug discontinuation and return to the patient's normal range in about seven to eight days [30, 38, 39].

## 3. Conclusion

Immune thrombocytopenia occurring as a response to GP IIb/IIIa inhibitors is well documented in the existing literature, including multiple studies and case reports [40–49] and is diagnosed clinically by excluding other causes of thrombocytopenia and documenting resolution of thrombocytopenia upon drug discontinuation. This adverse effect resulting from tirofiban and other GP IIB/IIIa inhibitors is particularly concerning, considering how widely they are used in modern cardiovascular medicine practice for treatment of NSTEMIs and during percutaneous coronary interventions. Life-threatening complications such as alveolar and gastrointestinal system hemorrhages may occur in the course of thrombocytopenia [50]. DITP resulting from GP IIb/IIIa inhibitors puts these patients at an added risk due to these complications, which is in turn compounded due to the effects of the many antiplatelet drugs used in concurrence with GP IIb/IIIa inhibitors. The treatment of DITP includes discontinuation of the causative drug; monitoring of platelet count recovery; or treatment of severe thrombocytopenia with glucocorticoids, IVIG, or platelet transfusions depending on the clinical presentation [27].

## Figures and Tables

**Table 1:** Results of the automated complete blood count tests.

Date, time	Platelet count (G/L)	WBC count (G/L)	Hb (g/dl)/Hct (%)
8/5/17, 1.54 pm	232	8	14.2/41.8
8/6/17, 7.32 am	195	6.3	13.0/38.1
8/7/17, 7.52 am	233	7.9	13.9/41.4
8/8/17, 6.53 am	6	10.5	11.3/32.8
8/8/17, 11.50 am	10	10.6	12.1/36.2
8/8/17, 7.52 pm	19	8.6	11.5/33.9
8/9/17, 5.00 am	24	9.3	11.4/33.9

WBC: white blood cell; Hb: hemoglobin; Hct: hematocrit.

**Table 2:** The 4Ts score is the sum of the values for each of the 4 categories.

The 4Ts scoring system			
4Ts category	2 points	1 point	0 points
Thrombocytopenia	Platelet count fall >50% and platelet nadir ≥20	Platelet count 30%–50% or platelet nadir 10–19	Platelet count fall <30% or platelet nadir <10
Timing of platelet count fall	Clear onset days 5–10 or platelet fall ≤1 day (prior heparin exposure within 30 days)	Consistent with days 5–10 fall, but not clear (e.g., missing platelet counts); onset after day 10; or fall ≤1 day (prior heparin exposure 30–100 days ago)	Platelet count ≤4 days without recent exposure
Thrombosis or other sequelae	New thrombosis (confirmed); skin necrosis; acute systemic reaction postintravenous unfractionated heparin bolus	Progressive or recurrent thrombosis; nonnecrotizing (erythematous) skin lesions; suspected thrombosis (not proven)	None
Other causes of thrombocytopenia	None apparent	Possible	Definite

Scores of 1–3, 4-5, and 6–8 are considered to correspond to a low (<5%), intermediate (∼14%), and high (∼64%) probability of HIT, respectively.
